# BK polyomavirus infection promotes growth and aggressiveness in bladder cancer

**DOI:** 10.1186/s12985-020-01399-7

**Published:** 2020-09-14

**Authors:** Yigang Zeng, Jiajia Sun, Juan Bao, Tongyu Zhu

**Affiliations:** 1 Department of Urology, Shanghai Public Health Clinical Center, Fudan University, Shanghai, 201508 China; 2 Shanghai Key Laboratory of Organ Transplantation, Shanghai, China

**Keywords:** BK polyomavirus, Bladder cancer, Cell growth, Cell aggressiveness

## Abstract

**Background:**

Recent studies have confirmed the integration of the BK polyomavirus (BKPyV) gene into the cellular genome of urothelial carcinomas in transplant recipients, further confirming the correlation between BKPyV and urothelial carcinomas after transplantation. However, the role BKPyV infections play in the biological function of bladder cancer remains unclear.

**Methods:**

We developed a BKPyV-infected bladder cancer cell model and a mice tumor model to discuss the role of BKPyV infections.

**Results:**

Our research proves that BKPyV infections promote the proliferation, invasion and migration of bladder cancer cells, while the activation of β-catenin signaling pathway is one of its mediation mechanisms.

**Conclusions:**

We first described BKPyV infection promotes the proliferation, invasion and migration of bladder cancer. We verified the role of β-catenin signaling pathway and Epithelial-Mesenchymal Transition effect in BKPyV-infected bladder cancer. These results provide meaningful information towards the diagnosis and treatment of clinical bladder cancer.

## Introduction

Urothelial carcinoma is one of the most common and highly malignant tumors in the urinary system. According to 2018 statistics from the American Cancer Society, bladder cancer is the sixth most common malignancy in males after lung, prostate, colorectal, stomach and liver [1]. Bladder cancer is also a prone tumor type for immunocompromised patients. According to research, kidney transplant recipients are three times more likely to have urothelial cancer than the general population [2, 3].

BK polyomavirus (BKPyV) is a human polyomavirus prone to reactivation in immunocompromised populations, especially transplant recipients. BKPyV reactivation after renal transplantation causes symptoms such as viremia, viruria, ureteral stricture and BKPyV-related nephropathy, as well as hemorrhagic cystitis after hematopoietic stem cell transplantation [4]. In 2012, at the International Agency for Research on Cancer (IARC) meeting, BKPyV and JCPyV were classified as “possibly” carcinogenic to human (group 2B) because of the “sufficient evidence” in experimental animals and the “inadequate evidence” in humans for their carcinogenicity [5]. So far, whether BKPyV has a causal role in the development of cancer was controversial. Early studies detected the presence of BKPyV large T antigens in prostate-, bladder- and kidney tumors [6–8], but several researchers thought BKPyV was unlikely to be involved in the etiology of most renal and bladder tumors because they had failed to detect BKPyV DNA in the cancer samples [9–11].

Many recent studies detected BKPyV gene integration in urinary tract epithelial cell tumors, providing further evidence for the correlation between BKPyV and urinary tract tumors [8, 12–17]. Interestingly, almost all of these integrations occurred in post-transplant tumors, but not in non-transplant tumors [17]. Therefore, BKPyV reactivation in immunosuppressed environments may be considered as a transforming factor leading to urothelial carcinomas. In addition, some studies have shown that transplant recipients with BKPyV viremia or polyomavirus-associated kidney disease have an increased risk (4–11 times) of bladder cancer when compared to transplant recipients without BKPyV [14, 18]. Although the underlying role of BKPyV in human cancers has not been fully determined, the close relationship between BKPyV and the development of urothelial cancer after transplantation, is without question. However, the functional effects of BKPyV infections on bladder cancer and the biological characteristics of tumor cells expressing BKPyV-related proteins have not yet been elucidated.

The Wnt/β-catenin pathway is implicated in cell proliferation and transcription, and regulating pattern formation during development. This pathway is involved in tumorigenesis and is associated with many biological behaviors such as tumor proliferation, invasion, and metastasis [19]. Besides, inhibition of β-catenin degradation in cytoplasm can induce epithelial-mesenchymal transition (EMT) and promote tumorigenesis [20]. Previous studies have shown that JCPyV large T antigen (LTag) binds directly to β-catenin, resulting in enhanced expression of β-catenin target genes such as c-myc and cyclin D1 [21–23]. BKPyV is thought to be oncogenic due to the expression of the early coding region-encoded proteins LTag and small T antigen (STag), which can initiate or drive neoplastic transformation. Whether constitutive activation of the Wnt/β-catenin signalling pathway by BKPyV LTag induce cancer remains to be established.

Therefore, this study explored the effects of BKPyV infections on the proliferation and migration of bladder cancer cells and the role of Wnt/β-catenin pathway and EMT in this process by establishing a model for BKPyV infections in bladder cancer cells and mice. Together, these results will clarify the characteristics of BKPyV-related bladder tumors, laying a foundation for further clinical trials.

## Materials and methods

### Cell culture

Human bladder cancer cell lines HTB-9 and T24 were obtained from the American Type Culture Collection (ATCC; Manassas, VA, USA) and cultured in RPMI 1640 (Gibco Life Technologies, Carlsbad, CA, USA) with 10% fetal bovine serum (FBS; Gibco Life Technologies) in a humidified atmosphere with 5% CO_2_ at 37 °C.

### BKPyV infection and inactivation

BKPyV stocks were initially propagated in Vero cells from viruses obtained from ATCC (VR-837, Dunlop). Viral lysates were made through three cycles of freezing the infected cells and supernatant at − 80 °C and thawing at 37 °C. The inactivation of BKPyV was performed at 100 °C for 10 min. Both T24 and HTB-9 cells were grown in culture dishes to 70% confluence, and infected with BKPyV. After infection for 2 hours, cells were washed three times with phosphate-buffered saline (Kaiji Co. Ltd., Shanghai, China), then were cultured using medium containing 2% serum with and without 15 μM KYA1797K (Med Chem Express, Princeton, NJ, USA), a potent and selective β-catenin inhibitor.

### Animal model

Male BALB/c nude mice (age 5 weeks, 18–20 g; Shanghai LC Laboratory Animal Co. Ltd., Shanghai, China) were housed in sterile filter-capped cages. Cultures of T24 and HTB-9, including their respective BKPyV infected cells (10^6^ cells in 100 mL PBS) were injected subcutaneously into nude mice with and without subsequent intraperitoneal injections of KYA1797K (25 mg/kg) (*n* = 5). Thirty days after implantation, tumors were surgically dissected and stored in liquid nitrogen before processing for histopathological examination. All animal experiments were performed according to the Guidelines for the Care and Use of Laboratory Animals and were approved by the Institutional Animal Care and Use Committee of Shanghai Public Health Clinical Center, Fudan University.

### Cell counting Kit-8 assay

The Cell Counting Kit-8 assay (CCK-8; Xiang SAM, Shanghai, China) was used to determine cell viability. Cells (2 × 10^3^ cells/well) were seeded in 96-well plates according to the manufacturer’s instructions. Absorbance of the medium at 450 nm was detected using a spectrophotometer by assessing cell viability. All observations were reproduced at least three times in independent experiments.

### Colony-formation assay

Cells were seeded in six-well plates at an initial density of 200 cells/well. Colonies were clearly visible after 10–14 days and selected cells were fixed with 4% paraformaldehyde for 30 min at room temperature and stained with 4 mg/mL crystal violet (Sigma-Aldrich). Colonies containing > 50 cells were counted using light microscopy (100×; Olympus, Tokyo, Japan). The average number of colonies was determined from three independent experiments.

### Cell migration and invasion assays

Transwell chambers (8-μm pore size; Corning, Corning, NY, USA) pre-coated with and without Matrigel (BD Biosciences) was used to determine cell invasion and migration. Cells (10^5^) in 200 μL FBS-free medium were seeded in the upper chamber, and 600 mL medium containing 10% FBS was added to the lower chamber. After several hours of incubation, cells that had migrated or invaded through the membrane were stained with methanol and 0.1% crystal violet solution. A light microscope (200×; Olympus, Tokyo, Japan) was used to count the number of cells in five random fields of view. The mean cell number was calculated for each group.

### Scratch wound healing assay

Cells were inoculated in six-well plates and cultured at 37 °C in a 5% CO_2_ cell incubator. After the cells reached 90% confluence, wounds of approximately 1 mm width were created using a sterile pipette tip. Cells were washed, incubated and continuously cultured in serum-free medium. Cultures at 0, 8, and 24 h were observed under an inverted microscope (40×; Olympus, Tokyo, Japan).

### Histological assessment

Hematoxylin and eosin(H&E) staining was performed for histological assessments. Tumor tissues were cut into small pieces and soaked in 4% neutral formaldehyde solution. Tissue blocks were washed with distilled water and stored in 70% ethanol overnight. After being dehydrated, embedded in transparent paraffin and sectioned, slides were sealed with polylysine and stained using H&E.

### Immunofluorescence staining

Cell slides and frozen sections in 4% formaldehyde (10% PFA, Polysciences, Eppelheim, Germany) were diluted in PBS for 10 min and permeabilized with 0.2% Triton X-100 (10%, Sigma-Aldrich) for 10 min at room temperature (RT). Fixed cells were blocked with blocking buffer containing milk powder and PBS for 15 min (37 °C). Primary and secondary antibodies were diluted in blocking buffer and incubated at RT for 50 min each. Primary antibodies included the polyclonal human rabbit anti-β-catenin (1:1000; Cell Signaling Technology, Beverly, MA, USA) and the monoclonal mouse anti-Large tumor antibody (1:50; PAb416, Abcam, Cambridge, MA, USA). Secondary antibodies used were the anti-mouse IgG1–Alexa Fluor 647 (1:800; Abcam), anti-rabbit IgG–Alexa Fluor 488 (1:1000; Abcam) and direct staining with Hoechst 33342 dye (1:1000; Abcam). Fluorescence was detected using inverted fluorescence microscope (EVOS XL Core, Thermo Fisher Scientific, USA). All images were processed using ImageJ software (NIH, Bethesda, MD, USA).

### Western blot

Western blotting was carried out as previously described [16]. Membrane proteins were separated on SDS-polyacrylamide gel electrophoresis and transferred to polyvinylidene difluoride membranes. Membranes were blocked using 5% milk and incubated overnight with anti-β-catenin, anti-cMYC, anti-Slug, anti-claudin-1 and anti-β-actin antibodies (1:1000; all from Cell Signaling Technology). Results were analyzed following previously described methods [24].

### Statistical analysis

All generated data is presented as mean ± standard deviation (SD). Statistical analyses were performed using the unpaired Student’s t-test or one-way Analysis of Variance (ANOVA) for more than two groups. Significant differences were considered when vales had *P* < 0.05. All analyses were carried out using Graph pad prism 7 software.

## Results

### BKPyV infection in bladder cancer cells is a non-lytic infection

**Fig. 1:**
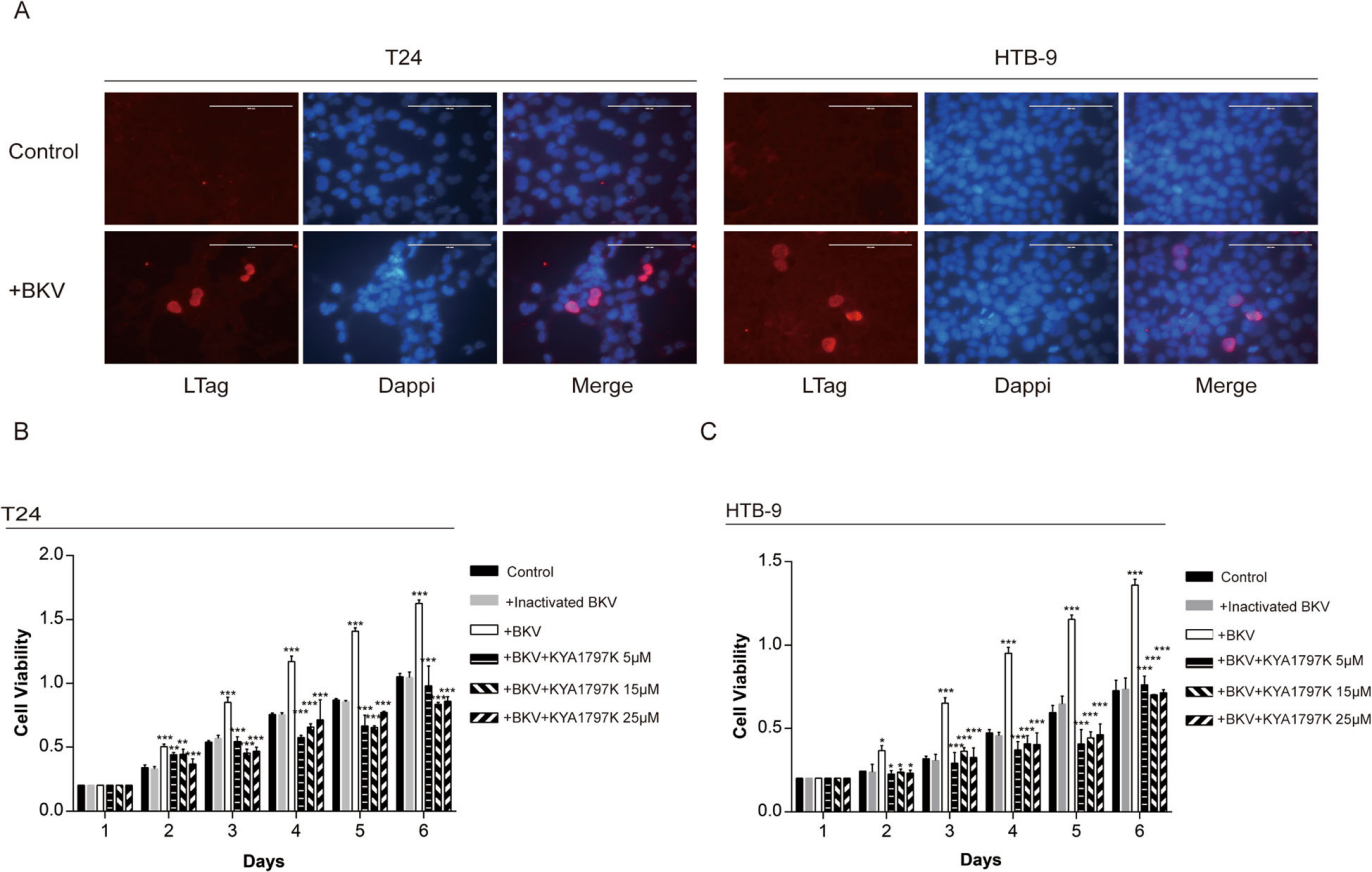
BKPyV infection in bladder cancer cells is a non-lytic infection (MOI 2; 48 h post infection). **a** Immunofluorescence staining was performed (red: LTag, blue, DNA, and images were taken at 400× magnification). **b**, **c** BKPyV infection significantly increased growth in T24 and HTB-9 cells, as measured by CCK-8. The effect was significantly reversed by KYA1797K. All graphs represent the mean ± SD obtained from three independent experiments. *P < 0.05, **P < 0.01, ***P < 0.001; + BKV versus control; + BKV + KYA1797K versus + BKV; Student’s t-test or one-way ANOVA

We used two bladder cancer cell lines, T24 and HTB-9, to infect with BKPyV. Forty-eight hours after infection, T24 and HTB-9 cells showed large T protein expression of BKPyV (Fig. 1a). By extending the infection time, we observed the effect of BKPyV infections on cell fates. Both T24 and HTB-9 cells did not lyse due to BKPyV infections, but continued to proliferate and grow for 9 days after the initial infection (Fig. 1b, c). Further research shows the BKPyV infected T24 and HTB-9 cells xenografted on mice still express large T antigen after 30 days (Fig. S1). Interestingly, VP1 expression in these two cell infection models increased continuously in the first few days, but maintained a stable level after that (Fig. S2).

### BKPyV infection promotes proliferation, invasion and migration of bladder cancer cells

**Fig. 2:**
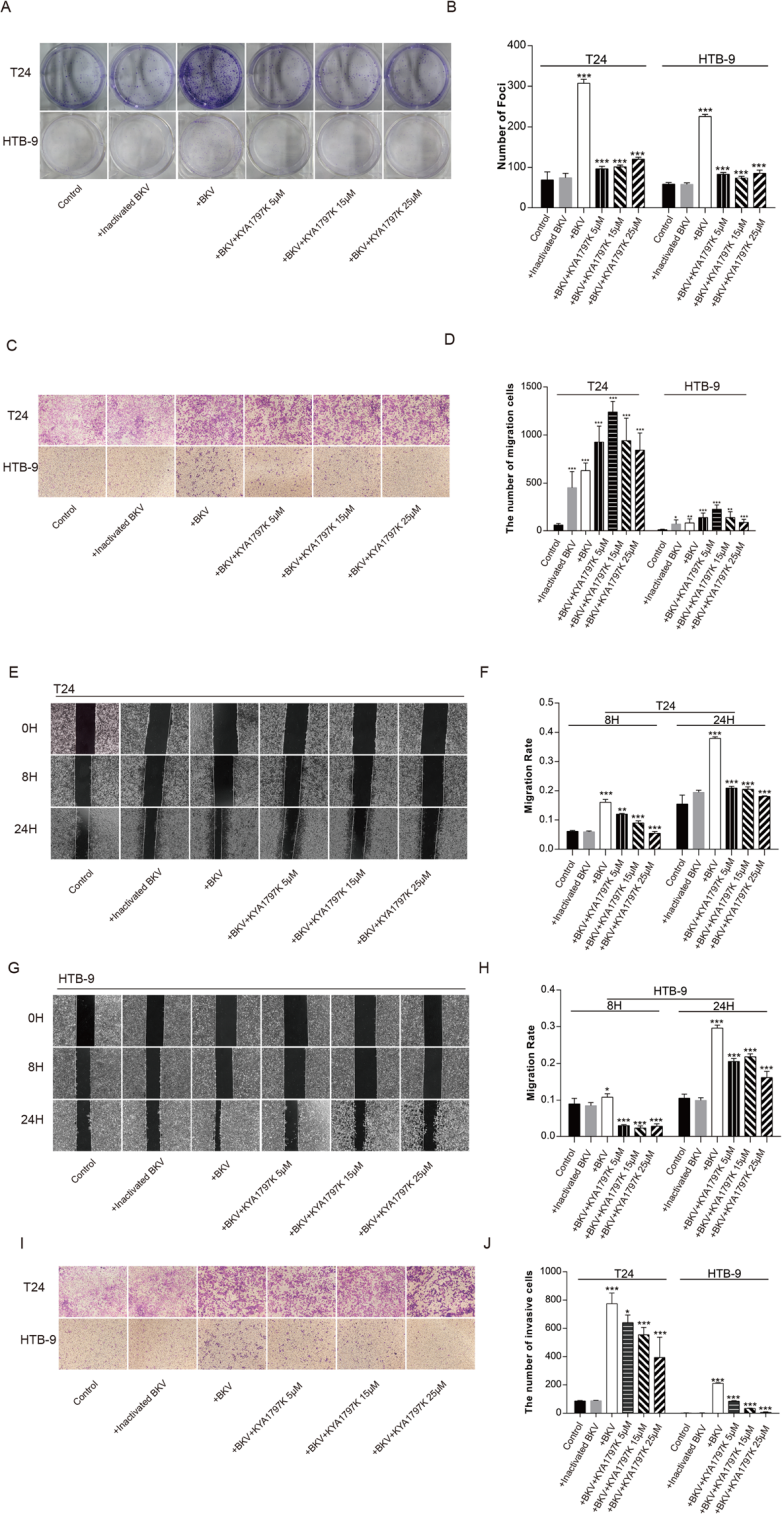
BKPyV infection promotes proliferation, invasion and migration of bladder cancer cells. **a**, **b** BKPyV infection significantly increased growth in T24 and HTB-9 cells, as measured by colony formation. **c**–**h** BKPyV infection significantly promoted migration of T24 and HTB-9 cells, as measured by the Transwell migration assay (**c**, **d**) and wound healing assay (**e**–**h**) after infected with BKPyV 48 h. **i**, **j** Transwell invasion assay showed that BKPyV infection significantly promoted invasiveness of T24 and HTB-9 cells. These effect was significantly reversed by KYA1797K. All images were taken at 100× magnification. All graphs represent the mean ± SD obtained from three independent experiments. *P < 0.05, **P < 0.01, ***P < 0.001; + BKV versus control; + BKV + KYA1797K versus + BKV; Student’s t-test or one-way ANOVA

Results from the CCK8 experiment showed that after infection by BKPyV, the cells proliferation ability and activity in both T24 and HTB-9 bladder cancer cells were significantly increased compared to cells not infected with BKPyV (inactivated BKPyV virus) (Fig. 1b, c). Colony formation experiments further verified our results, showing that BKPyV infections promote proliferation of bladder cancer cells in vitro (Fig. 2a, b). Transwell migration results showed that T24 and HTB-9 cells infected with BKPyV had significantly higher migration rates than the controls (Fig. 2c, d). Increased cell migration was most pronounced when multiplicity of infection is 2 (Fig. S3).

In addition, the scratch healing test demonstrated that BKPyV infected T24 and HTB-9 cells migrated significantly faster on plates than the control group (Fig. 2e–h). The two experiments consistently showed that BKPyV infection promoted the migration of bladder cancer cells in vitro. BKPyV infected T24 and HTB-9 cells had significantly higher invasive capacities than non-infected cells (Fig. 2i, j).

## BKPyV infection enhances bladder tumor growth and metastasis

**Fig. 3:**
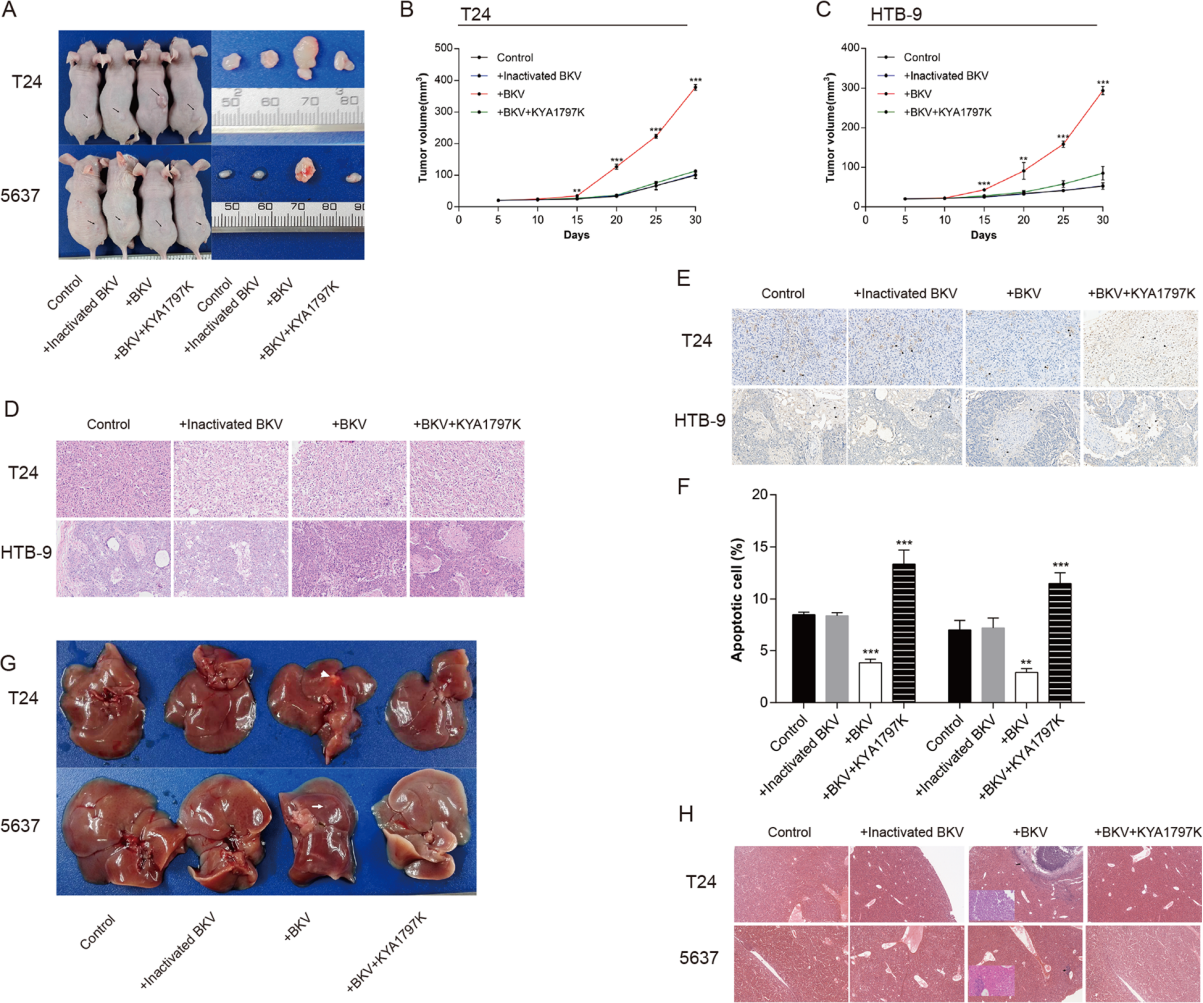
BKPyV infection enhances bladder tumor growth and metastasis. **a** T24, HTB-9 and their respective BKPyV-infected cells were injected subcutaneously into nude mice with subsequent intraperitoneal injection of KYA1797K (20 mg/kg) (*n* = 5). Tumor volumes (**b**, **c**) of mice were measured every 5 days. BKPyV infection significantly promoted tumor growth in vivo. The effect was significantly reversed by KYA1797K. Tumors were subjected to H&E staining (**d**) (200×) and TUNEL immunohistochemistry (**e**, **f**) (200×). TUNEL staining showed that BKPyV infection significantly induced tumor apoptosis. The effect was significantly reversed by KYA1797K. **g** Representative images of liver, metastatic nodules (indicated by arrows) are shown. **h** Livers were subjected to H&E staining (original magnification,40×; smaller image at lower left, 200×). Liver metastasis was found only in the BKPyV-infected group. The effect was significantly reversed by KYA1797K. All graphs represent the mean ± SD obtained from three independent experiments. *P < 0.05, **P < 0.01, ***P < 0.001; + BKPyV versus control; + BKPyV +KYA1797K versus + BKPyV; Student’s t-test or one-way ANOVA

The growth rate of xenografts of BKPyV-infected bladder tumor cells in mice increased significantly, with the tumor volume of the two cell lines being significantly larger than that of the control group at 15 days post inoculation. Differences in tumor volume became more pronounced over time (Fig. 3a–f). On day 30 post inoculation, mice were euthanized in order to observe tumor cell invasion and metastases. New tumors were discovered in the liver of BKPyV infected bladder tumor mice, while no tumor tissue was observed in other organs of the control mice (Fig. 3g).

Pathological morphology revealed that tumor tissue present in the livers were characteristic of urothelial carcinoma, identified as bladder tumor cells transported by the blood stream (Fig. 3h).

### BKPyV infection enhances β-catenin signaling pathway activation and epithelial-Mesenchymal transition (EMT) effect in bladder cancer cells

To explore the role of β-catenin signaling pathway in this process, we examined levels of β-catenin and its downstream signaling molecule cMYC in BKPyV infected tumor cells. We also examined changes in the expression of key molecules of EMT: Slug and Claudin-1.The Slug protein leads to the destruction of the inter-epithelial cell connection and promotes EMT; Claudin-1 is a tight junction protein whose down-regulation promotes cell dissociation, leading to epithelialization.

**Fig. 4:**
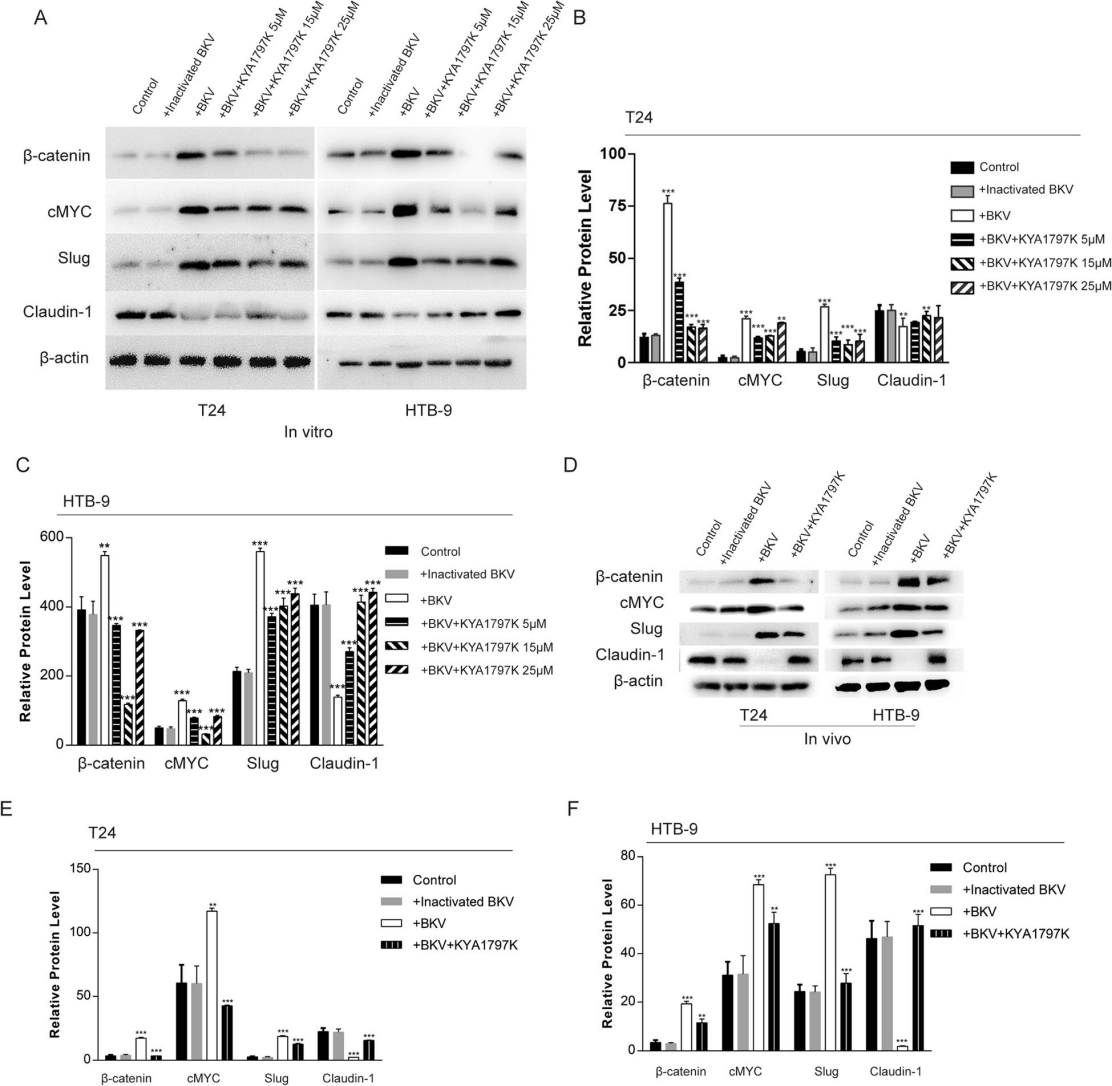
BKPyV infection enhances β-catenin signaling pathway activation and EMT effect in bladder cancer cells. **a**-**f** Expression levels of β-catenin, cMYC, and EMT-related proteins investigated by western blotting. β-Actin was used as a loading control. The effect was significantly reversed by β-catenin inhibitor (KYA1797K). All graphs represent the mean ± SD obtained from three independent experiments. *P < 0.05, **P < 0.01, ***P < 0.001; + BKPyV versus control; + BKPyV +KYA1797K versus + BKPyV; Student’s t-test or one-way ANOVA

In vitro, when compared to the control, the expression of β-catenin, cMYC and Slug proteins in BKPyV-infected cells were significantly increased, while the expression of Claudin-1 protein was significantly reduced (Fig. 4a–c).

In vivo, β-catenin protein expression in xenografts of BKPyV-infected bladder tumor cells were significantly higher than in the control (Fig. 4d–f). These results indicate that BKPyV infection promotes β-catenin signaling activation and Epithelial-Mesenchymal Transition (EMT) effects in bladder cancer cells.

### Blocking β-catenin signal inhibits BKPyV infection-mediated enhancement of proliferation and migration

Compared to BKPyV infected cells alone, BKPyV infected cells treated with KYA1797K had significantly reduced cell proliferation (Fig. 1b, c, 2a, b), migration (Fig. 2c, d) and invasion capacities (Fig. 2e, f). In vivo, expression levels of Slug were significantly reduced while expression of Claudin-1 was significantly increased (Fig. 4a–c). In vivo, BKPyV infection with KYA1797K applied showed significant reduction of tumor size and invasive abilities (Fig. 3a–f), with no metastasis occurring (Fig. 3g, h). Tumors showed significantly reduced Slug expression and significantly increased Claudin-1 expression (Fig. 4d–f).

## Discussion

Tumors have gradually become one of the main factors affecting the long-term survival of kidney transplant recipients. In recent years, the correlation between BKPyV infection and tumorigenesis in immunocompromised individuals has drawn increased attention. The idea that BKPyV plays an important role in cancers of the urinary system was recently confirmed by deep sequencing studies, which confirmed that the BKPyV gene was integrated into the genome of renal cancer and urothelial carcinoma cells after transplantation [13–15]. Besides, Querido et al. showed that JCpyV may cause urothelial carcinoma after transplant [25], which also supports the argument that human polyomavirus is closely related to bladder cancer.

When BKPyV infects cells, two different outcomes may occur: (1) the host cell allows replication of the virus, resulting in viral DNA amplification, progeny virion production and cell lysis; or, (2) the host prevents virus replication, and the persistent expression of the LTag causes abortion of infection cell or cell transformation. This result is determined by the continuous expression of LTag [26]. A small amount of LTag however is not sufficient to cause tumors. When BKPyV is activated in vivo, BKPyV triggers abnormally high expression of LTag in the host cell through various mechanism, eventually leading to cell transformation. Based on our results, BKPyV infections did not have a lytic effect on bladder cancer cells. It may be that these two tumor cell lines are already in an infinite proliferation state. A variety of cell cycle regulatory proteins mediate the continuous expression of BKPyV LTag in cells, leaving the virus unable to copy normally.

Previous research pointed out that LTag and STag of BKPyV can promote host cell transformation and immortalization, leading to enhanced cell proliferation capacity [27–29]. However, the role of BKPyV autoantigen expression in tumor cells has not been studied, and the biological characteristics of BKPyV-related tumors are unknown. With the help of the BKPyV infection tumor cell model, we were able to confirm that the proliferative capacity, migration and invasion ability of bladder tumors expressing BKPyV-related proteins were further increased in vitro. Their ability to grow, invade and metastasize to other nearby organs in vivo was highly likely, and is of further threat to clinical patients. After BKPyV infections, liver metastases of bladder tumors are likely to occur, which will increase the difficulty of clinical treatments and seriously affect the survival rates of patients.

It was previously found that LTag are prooncogenic due to their ability to inactivate tumor suppressor proteins, such as p53 and retinoblastoma protein (pRb), leading to increased cell proliferation [27, 28]. In addition, STag has been shown to increase activation of the mitogenactivated protein (MAP) kinase pathway, which may also augment cell proliferation and transformation [29].

Wnt/β-catenin signaling pathway plays an important role in tumor invasion and metastasis [19]. In the nucleus of tumor cells, β-catenin combined with the transcription factor family Tcf / Lefs can activate genes such as cMYC and cause cell proliferation and EMT effects [20]. JCPyV large T antigen can interact with β-catenin and stimulate expression of β-catenin target genes. We also found that BKPyV infections promotes β-catenin signaling pathway activation and EMT effects. Besides, blocking β-catenin signaling pathways can inhibit BKPyV’s function to promote tumor cell proliferation and migration invasion. These results suggest that β-catenin activation in these BKPyV-infected tumor cells may be related to the overexpression of LTag. Such activation of β-catenin further promotes the invasion and migration of tumor cells. At the same time, it can enhance the expression of cMYC to promote cell proliferation. This new discovery has considerable significance towards clinical treatments of infectious BKPyV bladder tumors. In our study, xenografts of BKPyV-infected bladder tumor cells are prone to distant metastasis. Blocking the β-catenin signaling pathway can inhibit this process, which may be an effective alternative for clinical treatment of BKPyV-infected bladder tumors.

## Conclusions

In summary, we first described BKPyV infection promotes the proliferation, invasion and migration of bladder cancer and bladder tumors expressing BKPyV-related proteins were more invasive in vitro. We verified the role of β-catenin signaling pathway and EMT effect in the characteristics of BKPyV-related bladder cancer. These results may help clinical diagnoses and treatment of BKPyV-related bladder cancer.

## Supplementary information

**Additional file 1: Figure S1.** Detection of large T antigen in BKPyV infected T24 and HTB-9 cells xenografted on mice after 30 days. BKPyV infected T24 and HB-9 cells xenografted on mice were obtained and made into paraffin sections.(A) Immunofluorescence staining was performed (red: LTag, green: β-catenin, blue: DNA, and images were taken at 100× magnification), and (B) In situ hybridization with LTag DNA probe was performed (cyan: LTag).

**Additional file 2: Figure S2.** Levels of VP1 protein and DNA in BKPyV-infected T24 and HTB-9 cells over time. T24 and HTB-9 cells were infected with BKPyV for 2,3, 6 and 9 days (A) Expression levels of VP1 proteins in cells was investigated by western blotting. β-tubulin was used as a loading control. (B) The relative level of VP1 DNA in cells was investigated by qRT-PCR. ACTB was used as a loading control. All graphs represent the mean ± SD obtained from three independent experiments. *P < 0.05, **P < 0.01, ***P < 0.001; Student’s t-test or one-way ANOVA.

**Additional file 3: Figure S3.** The most suitable BKPyV infection concentration. (A-B) Migration of T24 and HTB-9 cells was most pronounced when multiplicity of infection is 2, as measured by the Transwell migration assay. All graphs represent the mean ± SD obtained from three independent experiments. *P < 0.05, **P < 0.01, ***P < 0.001; + BKPyV versus control; Student’s t-test or one-way ANOVA.

## Data Availability

We can share our data if needed.

## Abbreviations

BKPyV/BKV: BK polyomavirus
ATCC: American Type Culture Collection
CCK-8: Cell Counting Kit-8 assay
H&E: Hematoxylin and eosin
RT: Room temperature
SD: Standard deviation
ANOVA: Analysis of Variance
EMT: Epithelial-Mesenchymal Transition
LTag: Large tumor antigen
STag: Small tumor antigen
pRb: Retinoblastoma protein
MAP: Mitogenactivated protein
