# Supplementary figures and images for: BK polyomavirus infection promotes growth and aggressiveness in bladder cancer
Source: Virol J. 2020 Sep 14;17:139. doi: 10.1186/s12985-020-01399-7 (PMC7488779; doi:10.1186/s12985-020-01399-7)

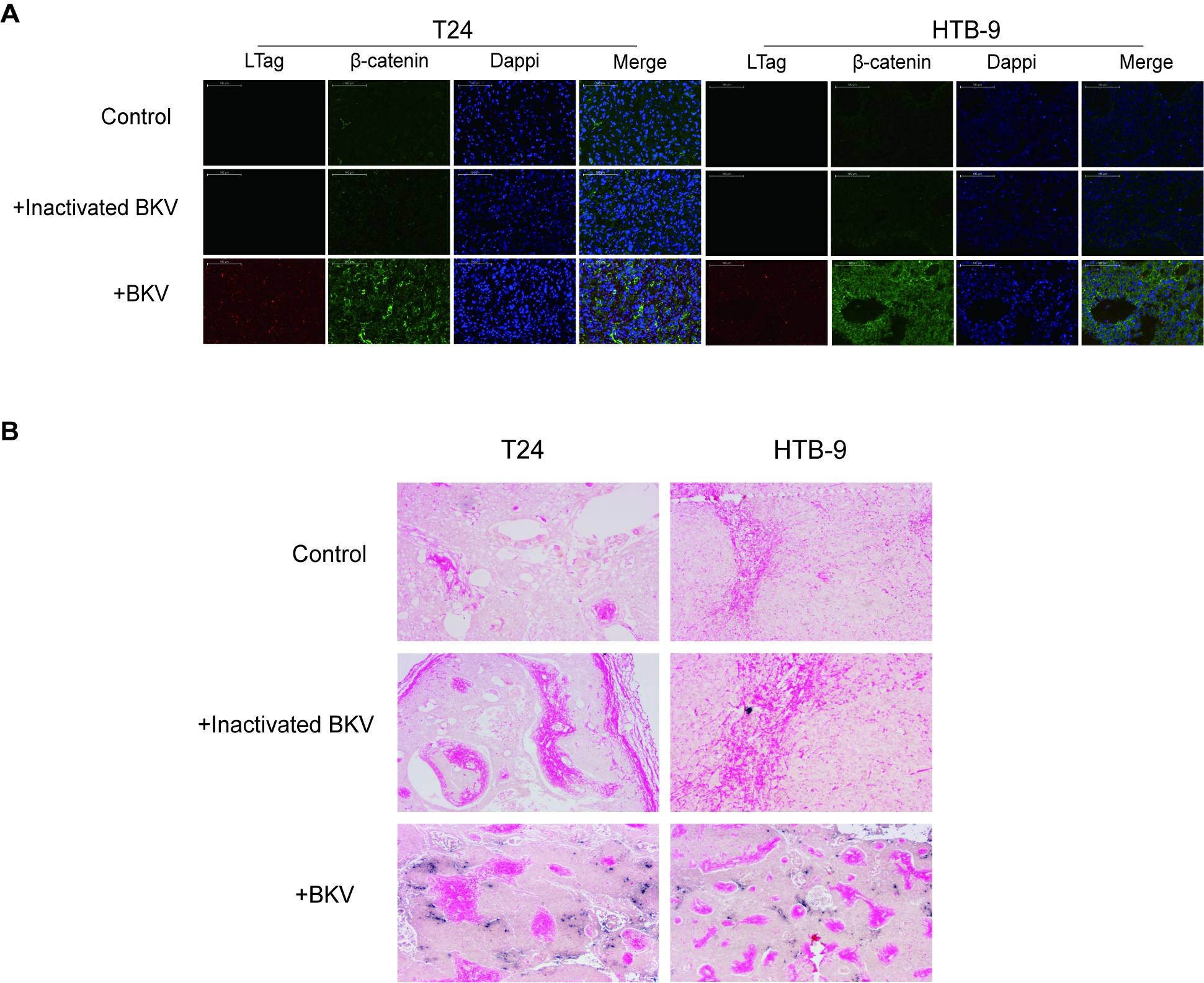

Supplement: Supplementary file 1 — Additional file 1: Figure S1. Detection of large T antigen in BKPyV infected T24 and HTB-9 cells xenografted on mice after 30 days. BKPyV infected T24 and HB-9 cells xenografted on mice were obtained and made into paraffin sections.(A) Immunofluorescence staining was performed (red: LTag, green: β-catenin, blue: DNA, and images were taken at 100× magnification), and (B) In situ hybridization with LTag DNA probe was performed (cyan: LTag). [file 12985_2020_1399_MOESM1_ESM.tif]

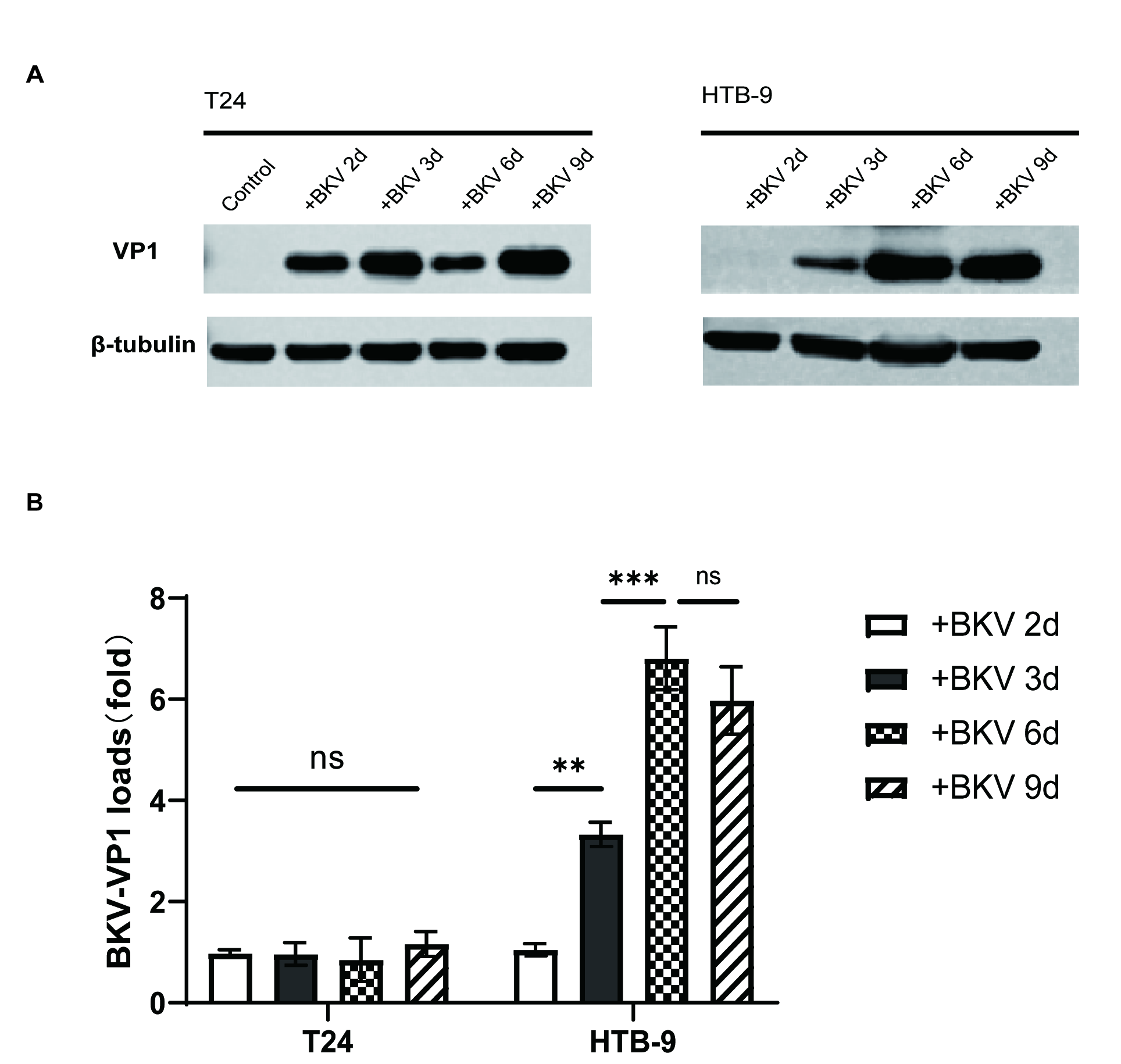

Supplement: Supplementary file 2 — Additional file 2: Figure S2. Levels of VP1 protein and DNA in BKPyV-infected T24 and HTB-9 cells over time. T24 and HTB-9 cells were infected with BKPyV for 2,3, 6 and 9 days (A) Expression levels of VP1 proteins in cells was investigated by western blotting. β-tubulin was used as a loading control. (B) The relative level of VP1 DNA in cells was investigated by qRT-PCR. ACTB was used as a loading control. All graphs represent the mean ± SD obtained from three independent experiments. *P < 0.05, **P < 0.01, ***P < 0.001; Student’s t-test or one-way ANOVA. [file 12985_2020_1399_MOESM2_ESM.tif]

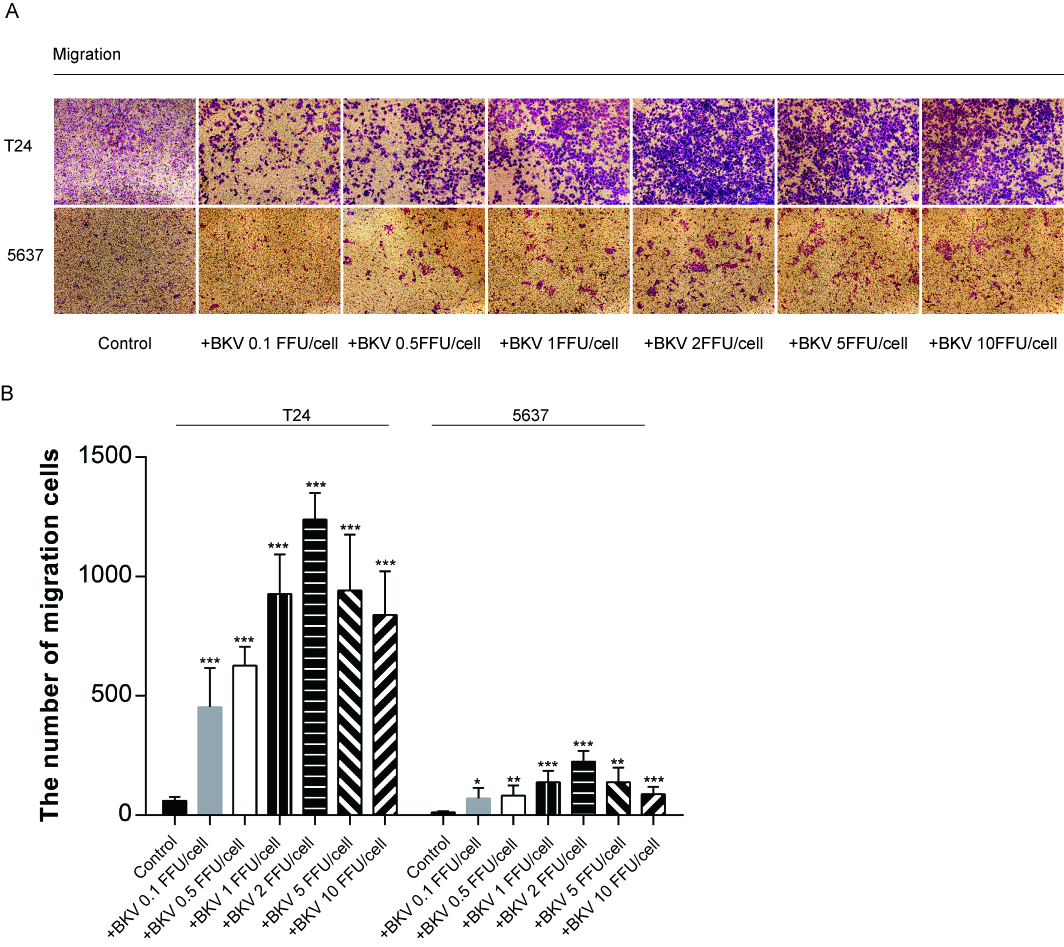

Supplement: Supplementary file 3 — Additional file 3: Figure S3. The most suitable BKPyV infection concentration. (A-B) Migration of T24 and HTB-9 cells was most pronounced when multiplicity of infection is 2, as measured by the Transwell migration assay. All graphs represent the mean ± SD obtained from three independent experiments. *P < 0.05, **P < 0.01, ***P < 0.001; + BKPyV versus control; Student’s t-test or one-way ANOVA. [file 12985_2020_1399_MOESM3_ESM.tif]
